# Clone-Node Detection in Wireless Sensor Networks Using Behavioral Analysis and Machine Learning

**DOI:** 10.3390/e28101137

**Published:** 2026-10-16

**Authors:** Swetha Pandithahalli Mahadevaswamy, Prasanna Bantaganahalli Thimmappa

**Affiliations:** Department of Computer Science and Engineering, JSS Science and Technology University, Mysuru 570006, Karnataka, India; swethapm@jssstuniv.in

**Keywords:** WSNs, clone detection, random forest, correlation-enhanced deep convolutional neural network (DCNN)

## Abstract

Wireless Sensor Networks (WSNs) remain vulnerable to clone-node attacks, in which malicious replicas use the identity or credentials of legitimate sensor nodes. This study presents an intelligent learning-based approach for clone-node detection using behavioral characteristics related to communication, topology, traffic, distance, and energy consumption. Since the WSN-DS dataset does not contain a native clone-node class, a controlled synthetic clone-node scenario was developed from legitimate sensor observations by preserving node identities while introducing behavioral changes associated with replication. A group-aware evaluation strategy was used to evaluate Logistic Regression, Random Forest, 1D-CNN, and a correlation-enhanced DCNN under the same experimental conditions. The results showed different detection characteristics among the evaluated models. Random Forest achieved the strongest overall classification performance, while Logistic Regression also provided strong discrimination with a low false-positive rate. The deep-learning models achieved high clone-detection recall, with the correlation-enhanced DCNN reaching 99.46% recall. These results demonstrate that different learning approaches provide different advantages for clone-node detection and highlight the importance of considering accuracy, detection sensitivity, and false-positive behavior together. The findings provide a useful basis for developing more reliable and adaptive intelligent clone-node detection methods for resource-constrained WSN environments.

## 1. Introduction

Wireless Sensor Networks (WSNs) are widely used to collect and exchange information in applications such as environmental monitoring, healthcare, industrial systems, surveillance, and smart infrastructure. A typical WSN consists of a large number of sensor nodes with limited energy, memory, processing, and communication resources [1]. Since these nodes are often deployed in unattended or difficult-to-access environments, they can be exposed to different security threats. Intrusion-detection mechanisms are therefore important for identifying abnormal or malicious activities within sensor networks [2].

Among these security threats, clone-node or node-replication attacks are particularly difficult to detect. In this type of attack, an adversary compromises a legitimate sensor node, obtains its identity or credentials, and introduces one or more replicas using the same identity. Distributed detection techniques have been proposed [3] to detect conflicting identity and location information associated with replicated nodes. The problem is harder in mobile conditions, where the legitimate nodes can naturally switch locations over time. Therefore, mobility aware techniques have been studied to distinguish normal node mobility from possible replication attacks [4]. Random-walk based approaches have been proposed to distribute the detection process over the network, decreasing its dependence on a centralized mechanism [5]. The effectiveness of clone-node detection depends on network topology, communication requirements, mobility, and the information available to the detection mechanism, as shown in previous studies [6].

Another approach to detect abnormal sensor behavior is the increasing use of data-driven security methods. The WSN-DS dataset was created to support research on intrusion detection using communication and operational information collected from WSN environments [7]. The detection of clone-nodes has also been studied using learning-based approaches. For example, deep-learning and optimization-based methods have combined network features and learned representations to detect replicated nodes [8].

In designing security mechanisms for WSNs, efficient use of resources and communication reliability are also important. Guru et al. [9] investigated one-hop forwarding for QoS routing and an enhanced fault-tolerant clustering mechanism for multimedia sensor networks. Their work highlights the need for reliable communication and clustering in resource-constrained sensor environments. These considerations are relevant to security applications because additional monitoring and detection processes should operate without placing unnecessary communication or energy demands on the network. More broadly, hybrid optimization with TensorFlow-based learning has also been used for computation-intensive decision problems, illustrating how learned models can be combined with optimization when the feature–decision relationship is complex [10].

Recent work in Entropy emphasizes that intrusion-detection performance depends strongly on the quality, relevance, and selection of network features [11], while newer entropy-assisted malicious-traffic detection studies continue to combine feature evaluation with machine learning and deep learning [12]. The characteristics of the available data can also influence the choice of learning algorithm. Deep-learning methods do not necessarily outperform conventional machine-learning approaches on every structured or tabular dataset [13]. For this reason, clone-node detection should be evaluated using different learning models rather than assuming that a more complex architecture will always provide better performance. Model evaluation should also consider more than overall accuracy. Precision-recall analysis can provide useful information about the balance between identifying malicious observations and incorrectly flagging legitimate ones [14]. Similarly, Principal Component Analysis (PCA) provides a useful way to examine multidimensional observations and understand their distribution in a reduced feature space [15].

Deep-learning models can nevertheless capture complex and nonlinear relationships among behavioral characteristics. Optimization methods such as Adam provide an efficient mechanism for updating model parameters during neural-network training [16]. At the same time, WSN applications must consider the energy required for communication and computation. Energy-efficiency mechanisms have therefore remained an important area of research for improving the operational lifetime and resource utilization of sensor networks [17].

Based on these considerations, this study investigates intelligent clone-node detection using behavioral analysis and multiple learning approaches. Logistic Regression, Random Forest, 1D-CNN, and correlation-enhanced DCNN are evaluated under the same experimental conditions. Communication, topology, traffic, distance, and energy-related characteristics are considered together to examine the different detection characteristics and performance trade-offs of the evaluated models.

For experimental evaluation, legitimate sensor observations are obtained from the WSN-DS dataset [7]. Since WSN-DS does not contain a native clone-node attack class, synthetic clone-node observations are generated from legitimate records. The original node identities are preserved while selected cluster-head, communication-distance, traffic, packet, and energy characteristics are modified to represent behavioral inconsistencies associated with node replication. A group-aware evaluation strategy is used to prevent related legitimate and synthetic clone-node observations from being separated across the training and testing partitions.

The main contributions of this work are as follows:

1. A clone-node detection framework is developed for hybrid WSN environments by combining behavioral analysis, feature correlation, and deep convolutional learning.
2. A synthetic clone-node attack scenario is constructed from legitimate WSN-DS sensor observations while preserving replicated node identities and introducing communication, topology, traffic, and energy inconsistencies associated with clone behavior.
3. A common behavioral feature representation and correlation analysis are used to examine relationships among sensor characteristics and support the evaluated learning algorithms.
4. The framework is evaluated using multiple measures, including accuracy, precision, recall, F1-score, ROC-AUC, false-positive rate, confusion-matrix analysis, and decision-threshold behavior, together with conventional machine-learning and neural-network baselines.
5. Additional behavioral, correlation, and feature-space analyses are used to examine how legitimate and synthetic clone-node observations differ and to provide a more transparent interpretation of the detection process.

The remainder of this paper is organized as follows. Section 2 reviews existing clone-node detection techniques and related learning-based approaches. Section 3 describes the proposed framework, clone-node attack construction, feature processing, and the evaluated models. Section 4 presents the experimental results, comparative analysis, and computational considerations. Section 5 presents the conclusions, and Section 6 outlines future research directions.

## 2. Literature Review

Recent research on clone-node detection has considered both static and mobile WSN environments. In mobile networks, changes in node location do not necessarily imply malicious activity, since legitimate nodes may naturally move through the network. Cheng et al. [18] suggested a multi-point collaboration protocol for mobile replica-node detection based on key negotiation and cryptographic mechanisms. Their method improves replica detection with lower communication, storage and energy overhead in mobile WSNs.

Security is an important issue in WSNs and it also impacts the efficiency of communication. Praveen et al. [19] studied the energy efficient communication using an auto-associative polynomial convolutional neural network. Their work indicates how learning-based techniques can be applied to communication and resource-management problems in sensor networks. Such considerations are relevant to security systems, since additional monitoring and classification should work under the limited energy and communication resources of the sensor nodes.

Several techniques have also been proposed for clone-node detection. Numan et al. [20] proposed a hybrid method to identify clone nodes in static WSNs. This method showed that it is possible to use multiple detection mechanisms together instead of relying on just one indicator. Alkhafagee and Alsharifi [21] studied node-clone detection protocols and different approaches to protect WSNs from replication attacks. In their study, they concentrate on the importance of network structure, communication overhead, detection strategy and allocation of resources for the design of clone-detection mechanisms.

Intrusion-detection research offers a broader scope for malicious-behavior detection in WSNs. In a review of intrusion-detection systems for sensor networks, Butun et al. [2] discussed the challenges of identifying abnormal network activities while operating under limited computational and communication resources. These challenges are also relevant to clone-node detection, because a compromised node may have valid credentials but may show communication or operational behavior that is different from legitimate sensor activity.

Energy efficient routing is another significant challenge in resource constrained sensor networks. Ananda Babu et al. [22] proposed an energy-efficient routing protocol using RD-MAC for WSNs. Their work emphasizes the importance of reducing communication and energy requirements during network operation. This is also relevant when security mechanisms are introduced, since continuous behavioral monitoring and classification may increase the workload of sensor or edge devices.

Feature selection and relationships among network characteristics can also influence learning-based detection. Li et al. [23] investigated correlation-based feature subset selection, demonstrating the usefulness of identifying informative features while reducing unnecessary or redundant information. This is relevant to the present study because clone-node behavior is examined through relationships among communication, topology, traffic, distance, and energy-related characteristics rather than through a single sensor attribute.

The structure of WSN telemetry also makes model selection important. Shwartz-Ziv and Armon [13] showed that deep learning does not necessarily provide better performance than conventional machine-learning approaches for tabular data. WSN behavioral observations are similarly represented through structured numerical characteristics. It is therefore useful to compare conventional models such as Logistic Regression and Random Forest with convolution-based neural-network approaches rather than assuming beforehand that deep learning will provide the strongest classification performance.

Evaluation measures are equally important when comparing these approaches. Saito and Rehmsmeier [14] discussed the value of precision-recall analysis for binary classification, particularly when the relationship between successful detection and false alarms needs to be examined. This consideration is relevant in clone-node detection, since high attack recall may still be associated with a large number of legitimate nodes being misclassified as malicious.

As an alternative, deep neural networks learn increasingly complex representations of input characteristics [24]. This ability makes them useful for analyzing non-linear relationships in multidimensional data. However, their effectiveness depends on the structure and quality of the features available. Learning models combined with optimization strategies can also be beneficial for computational learning problems. Chowlur Revanna and Al-Nakash [10] illustrated how to apply a model based on TensorFlow with hybrid optimization to solve a computation-intensive decision task.

In this study, we make use of the literature reviewed to evaluate Logistic Regression, Random Forest, 1D-CNN, and correlation-enhanced DCNN on the same clone-node dataset and experimental conditions. Instead of trying to say one model is better than the other, the study compares the models based on their ability to distinguish the difference in behavior between legitimate and synthetic clone-node observations. This comparative approach gives a better insight into the trade-offs between the overall classification performance, the sensitivity of clone detection and the false positive behavior.

## 3. Methodology

### 3.1. Datasets Description and Preprocessing

Experimental evaluation was conducted using the WSN-DS dataset, which includes communication and operational records gathered from wireless sensor nodes in different network conditions. The data set comprises data about node identity, cluster-head communication, transmission and reception activity, distance and energy consumption. These properties lay a good foundation for studying behavioral changes caused by the replication of nodes.

Since WSN-DS does not contain a native clone-node class, this study constructs a controlled synthetic clone-node scenario from 12,000 legitimate WSN-DS records; hereafter, the term synthetic clone-node is used consistently throughout the manuscript. Each legitimate record is paired one-to-one with a synthetic clone-node observation, producing 24,000 observations. The copied node identity is preserved while operational behavior is perturbed across multiple independent properties. Cluster-head association is reassigned with a probability of 0.72. Distance to the cluster head is multiplied by a factor selected from {0.35,0.55,1.45,1.80,2.25} and Gaussian noise N(0,3.0) is added. Time is shifted by an integer from −4 to +4. DATA_Sis scaled by a factor sampled uniformly from 1.25–2.40 with additive N(2.0,3.0) noise; DATA_R is scaled by 0.60–1.55 with N(0,2.0) noise; and Data_Sent_To_BS is scaled by 1.10–2.20 with N(1.0,2.5) noise. ADV, JOIN, and SCH send/receive counts receive binomial increments B(2,0.35). Expended Energy is scaled by 1.20–2.00 with N(0,0.015) noise, sender code is reassigned with probability 0.50, and cluster-head role is toggled with probability 0.12. Non-negative constraints are applied where appropriate. These multi-property perturbations create behavioral inconsistencies associated with identity replication without introducing a single deterministic clone marker. Because WSN-DS does not provide an independently observed clone-attack class, these records are used as a controlled, behaviorally motivated experimental representation of clone-node behavior. They are not treated as equivalent to measurements collected from confirmed clone attacks in a physical WSN, and the study therefore does not use them to claim real-world external validity.

Additional behavioral features were derived from the WSN-DS attributes, including transmission/reception totals and ratios, control-to-data ratio, energy per transmission, base-station forwarding ratio, distance–energy interaction, cluster-head identity gap, traffic–energy load, and cyclic time features. Pearson correlations are calculated using the training partition only. The eight strongest absolute-correlation feature pairs are selected, and for each selected pair (a,b) two relationship features are added: the product a×b and the absolute difference |a−b|. This produces the correlation-enhanced input representation. For the ablation comparison, the plain 1D-CNN receives only the standardized base behavioral features, whereas the correlation-enhanced DCNN receives the base features plus these correlation-derived relationship features. Both neural models use the same architecture: Conv1D (32, kernel 3), batch normalization, max pooling, Conv1D (64, kernel 3), batch normalization, global-average pooling, a 64-unit dense layer, 0.30 dropout, and sigmoid output. Both use Adam with a learning rate of 0.001, binary cross-entropy, a maximum of 18 epochs, batch size 512, and validation-loss early stopping with patience 3. Thus, the comparison isolates the effect of correlation-derived input augmentation while retaining the same neural architecture and training configuration. The engineered behavioral variables are tabular and do not possess a natural sequential ordering. Consequently, the convolutional models are evaluated here as exploratory comparative architectures rather than on the assumption that local one-dimensional convolution is intrinsically appropriate for unordered tabular WSN features. The feature-order sensitivity analysis described below is used to test this architectural limitation explicitly.

The final dataset contained two classes: legitimate nodes (0) and synthetic clone-nodes (1). To prevent pair-level and node-identity leakage, partitioning was performed using sensor-node identity (id) as the grouping variable. Consequently, all observations associated with a given node identity, including the legitimate observations and their derived synthetic clone-node counterparts, were retained within a single partition. The group-aware procedure targeted an approximately 70%/15%/15% train/validation/test division at the node-group level. Because complete node-identity groups could not be divided across partitions, the realized observation counts were 16,622 (69.26%) for training, 3706 (15.44%) for validation, and 3672 (15.30%) for independent testing. The independent test partition contained 1836 legitimate-node and 1836 synthetic clone-node observations and comprised node identities held out from model training. Numerical features were standardized using parameters obtained only from the training data. The processed data were then used to train and evaluate Logistic Regression, Random Forest, 1D-CNN, and the proposed correlation-enhanced DCNN under the same experimental conditions.

### 3.2. Additional Robustness and Sensitivity Analyses

Three supplemental analyses were performed to address stability, feature-order dependence, and dependence on the exact synthetic-generation rules. First, the complete experiment was repeated using five independent seeds (42, 52, 62, 72, and 82). For each seed, the synthetic generation, node-group partitioning, model fitting, and evaluation were repeated, and the mean and standard deviation of the principal metrics were calculated.

Second, because the 1D-CNN operates on an engineered tabular feature vector rather than a naturally ordered temporal sequence, a feature-order sensitivity analysis was performed on the fixed seed-42 partition. The original feature order was retained as a baseline and five fixed random permutations of the same input columns were evaluated using the identical 1D-CNN architecture and training settings. Only the order of the input columns was changed; feature values, observations, labels, partition membership, architecture, and optimization settings were held constant.

Third, an alternate-generator robustness test was conducted to examine whether the learned decision functions depended only on the exact perturbation parameters used in the primary synthetic generator. All models were fitted using only the original seed-42 generator, including training-only scaling and correlation selection, and were then evaluated on the same held-out node identities with clone observations regenerated under a separately parameterized, milder perturbation regime. The alternate regime reduced cluster-head reassignment from 0.72 to 0.40, used distance factors {0.70,0.85,1.20,1.35,1.55} with noise standard deviation 1.5, reduced the time shift to −2 to +2, narrowed the traffic and energy multipliers, reduced the control-count increment to B(1,0.20), reduced sender-code reassignment from 0.50 to 0.25, and reduced cluster-head-role toggling from 0.12 to 0.06. No alternate-generator observations were used for fitting, scaling, correlation selection, or validation. The purpose of this analysis is deliberately limited: it tests whether the fitted decision functions depend exclusively on the exact numerical parameterization of the original synthetic generator. Because the alternate observations are produced by the same general class of behaviorally motivated synthetic perturbation mechanism, this test does not establish external validity for real-world clone attacks. Validation on independently collected clone-attack observations or a physical WSN testbed is outside the evidence provided by the present dataset and remains necessary before real-world generalization can be claimed.

### 3.3. Flowchart of Proposed Work

**Figure 1:**
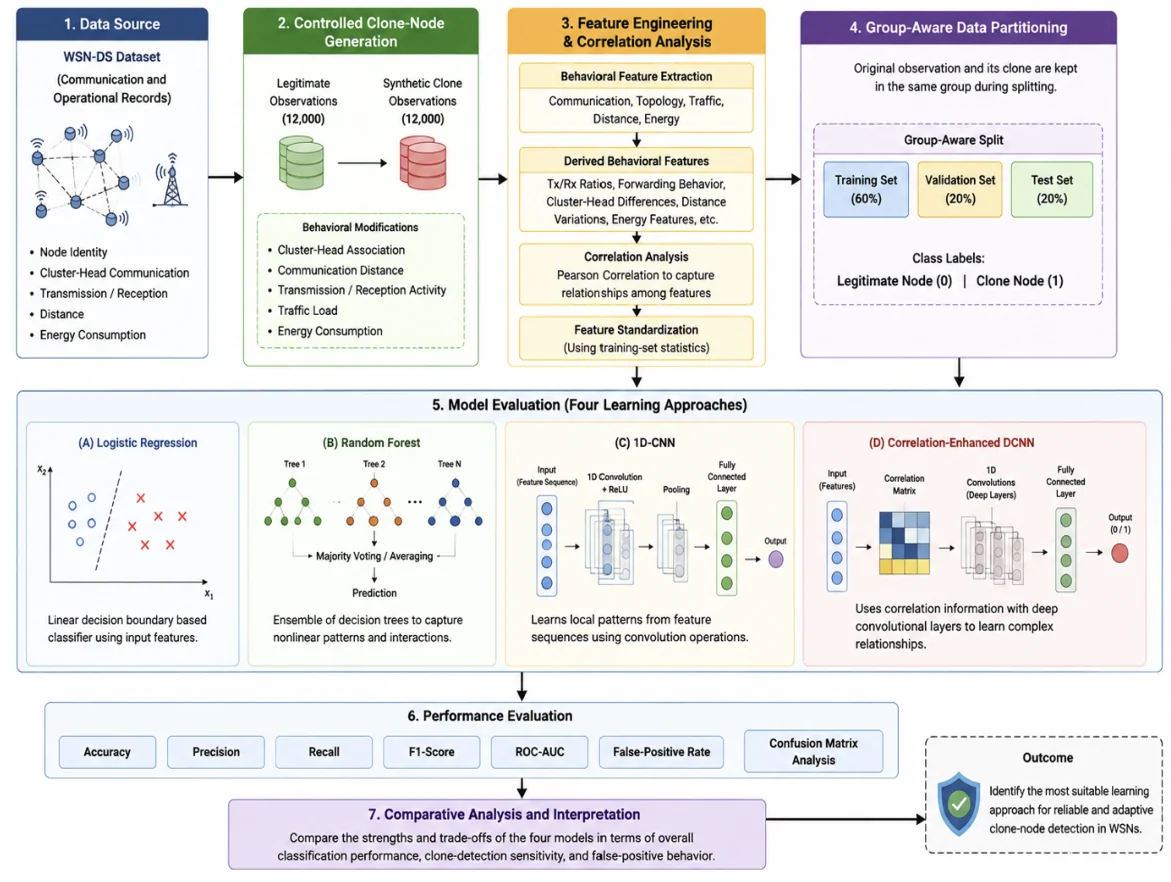
Overall framework for clone-node detection.

The proposed workflow for intelligent clone-node detection is illustrated in Figure 1. The process begins with legitimate sensor observations obtained from the WSN-DS dataset. Because of the absence of a specific clone-node attack class in the original dataset, synthetic clone-node observations are generated by retaining the identity of legitimate sensor nodes and injecting behavioral changes such as the cluster-head association, communication distance, packet transmission and reception, traffic activity, and energy consumption.

The relevant behavioral features are then extracted to represent communication, topology, traffic, distance, and energy behavior after building the experimental dataset. Then correlation analysis is performed to analyze the relationships between these characteristics. The numerical features are normalized before training the model. To mitigate information leakage, a group-aware data-partitioning strategy is adopted so that both the legitimate observation and the clone observation derived from it are in the same training, validation, or test partition.

The processed data are assessed under four learning approaches; Logistic Regression, Random Forest, 1D-CNN and correlation-enhanced DCNN. Multiple models enable comparison of traditional machine-learning and deep-learning methods under identical experimental conditions rather than depending on a single classifier. The model is evaluated using accuracy, precision, recall, F1-score, ROC-AUC, false-positive rate, and confusion-matrix analysis. This comparison is utilized to study strengths and trade-offs of the different methods to detect clone-node behavior in WSN environments.

### 3.4. Learning Models and Mathematical Formulation

Four classification approaches are evaluated in this study: Logistic Regression, Random Forest, a one-dimensional Convolutional Neural Network (1D-CNN), and a correlation-enhanced Deep Convolutional Neural Network (DCNN). The same processed behavioral dataset and group-aware training, validation, and test partitions are used for all models so that their detection performance can be compared under consistent experimental conditions. Logistic Regression and Random Forest provide conventional machine-learning baselines, while 1D-CNN and the correlation-enhanced DCNN represent deep-learning approaches. Because the input variables form an engineered tabular vector rather than a naturally ordered signal, the convolutional models are treated as exploratory comparative models in this study. Their inclusion permits direct examination of whether local convolution is useful under this representation, while the permutation analysis tests the extent to which their behavior depends on arbitrary feature adjacency.

The mathematical expressions below describe the main operations used in the learning framework. They are included to clarify the decision process of the conventional models and the feature processing and classification stages used by the neural models. The equations do not imply that one model is assumed to be superior; the relative performance of all four approaches is determined experimentally.

#### 3.4.1. Logistic Regression

Logistic Regression is used as a conventional linear baseline. For an input behavioral feature vector *x*, the probability that an observation belongs to the clone-node class is estimated using the logistic function:

(1) $$p\left(y=1|x\right)=\frac{1}{1+\exp(-\left(w^{T}x+b\right))}$$

where *x* is the input feature vector, *w* is the learned weight vector, *b* is the bias term, and p(y=1∣x) represents the estimated probability of the clone-node class. Using a decision threshold τ, the predicted class is:

(2) $$\hat{y}=1,\;\mathrm{if}\;p\left(y=1|x\right)\geq \tau ;\;\hat{y}=0,\;\mathrm{otherwise}$$

In the experiments, class 0 represents a legitimate observation and class 1 represents a synthetic clone-node observation. The default classification threshold is τ=0.50 unless threshold behavior is being examined separately.

#### 3.4.2. Random Forest

Random Forest provides an ensemble-based conventional machine-learning comparison. It constructs multiple decision trees using sampled training observations and randomly selected subsets of features. For classification, the final class is obtained from the collective decisions of the individual trees:

(3) $$\hat{y}=\mathrm{mode}\left\{h_{1}\left(x\right),h_{2}\left(x\right),\ldots ,h_{T}\left(x\right)\right\}$$

where $h_{t}\left(x\right)$ is the class predicted by the *t*-th decision tree and *T* is the total number of trees. The majority-voting mechanism allows the model to combine multiple nonlinear feature partitions rather than relying on a single decision tree.

#### 3.4.3. Behavioral Correlation Analysis

Correlation analysis is used to examine relationships among the behavioral characteristics in the experimental data. Let $X\in R^{N\times D}$ denote the telemetry matrix containing *N* observations and *D* behavioral features. The Pearson correlation coefficient between features *i* and *j* is calculated as:

(4) $$R_{ij}=\frac{\sum_{k=1}^{N}\left(x_{ki}-{\bar{x}}_{i}\right)\left(x_{kj}-{\bar{x}}_{j}\right)}{\sqrt{\sum_{k=1}^{N}{\left(x_{ki}-{\bar{x}}_{i}\right)}^{2}}\sqrt{\sum_{k=1}^{N}{\left(x_{kj}-{\bar{x}}_{j}\right)}^{2}}}$$

where $x_{ki}$ and $x_{kj}$ are the values of features *i* and *j* for observation *k*, while ${\bar{x}}_{i}$ and ${\bar{x}}_{j}$ are their corresponding sample means. The resulting correlation matrix $R\in R^{D\times D}$ is used to examine feature relationships and to support the correlation-enhanced representation used by the DCNN. The same behavioral feature set remains available to the other evaluated classifiers.

#### 3.4.4. 1D-CNN Feature Extraction

For the deep-learning models, the standardized behavioral feature vector is treated as a one-dimensional feature sequence. A 1D convolutional layer applies learned filters across this sequence to produce intermediate feature maps. The convolutional operation at layer *l* can be represented as:

(5) $$y_{c}^{l}=\sigma \left(\sum_{f}z_{f}^{l-1}*w_{c,f}^{l}+b_{c}^{l}\right)$$

where $z_{f}^{l-1}$ is the input feature map from the previous layer, $w_{c,f}^{l}$ is the convolutional filter connecting input channel *f* to output channel *c*, $b_{c}^{l}$ is the bias term, ∗ denotes the one-dimensional convolution operation, and σ(·) represents the nonlinear activation function. ReLU activation is used in the convolutional layers.

#### 3.4.5. Correlation-Enhanced DCNN Classification

The correlation-enhanced DCNN receives the standardized base behavioral features together with the correlation-derived product and absolute-difference features selected from the eight strongest training-set correlation pairs. The plain 1D-CNN receives the standardized base behavioral features without these added relationship features. Both variants use the same convolutional architecture and training configuration, allowing the effect of correlation-derived input augmentation to be evaluated directly. After the convolutional, pooling, dropout, and dense processing stages, the final neuron uses a Sigmoid activation function to estimate the probability of clone-node behavior:

(6) $${\hat{y}}_{p}=\frac{1}{1+\exp(-\left(w^{T}h+b\right))}$$

where *h* represents the high-level feature representation produced by the network, *w* and *b* are the parameters of the output layer, and ${\hat{y}}_{p}\in \left[0,1\right]$ is the predicted clone-node probability.

The final class is obtained using:

(7) $$\hat{Y}=1,\;\mathrm{if}\;{\hat{y}}_{p}\geq 0.50;\;\hat{y}=0,\;\mathrm{otherwise}$$

This threshold converts the predicted probability into a binary class label. Threshold-sensitivity analysis is also used in the experimental section to examine the balance between clone-node recall and false-positive behavior.

#### 3.4.6. Model Training Objective

The neural-network classifiers are trained as binary classification models. Binary cross-entropy is used to measure the difference between the true class label $y_{i}$ and the predicted probability ${\hat{y}}_{i}$ for each observation

(8) $$L_{BCE}=-\frac{1}{N}\sum_{i=1}^{N}\left[y_{i}\ln\left({\hat{y}}_{i}\right)+\left(1-y_{i}\right)\ln\left(1-{\hat{y}}_{i}\right)\right]$$

where *N* is the number of training observations, $y_{i}\in \left\{0,1\right\}$ is the true class label, and ${\hat{y}}_{i}$ is the predicted probability. Model parameters are updated during training to minimize this loss. Validation performance and early stopping are used to reduce unnecessary training once the validation loss no longer improves.

#### 3.4.7. Computational Requirement of 1D Convolution

For a 1D convolutional layer, the approximate computational requirement depends on the input sequence length, kernel size, number of input channels, and number of output filters. The dominant number of convolutional operations can be expressed approximately as:

(9) $$O(L\times K\times C_{in}\times C_{out})$$

where *L* is the input length, *K* is the convolution kernel size, $C_{in}$ is the number of input channels, and $C_{out}$ is the number of output channels. This expression is a high-level indication of computational growth, instead of a hardware specific latency measurement. Actual execution cost is also a function of memory access, parameter storage, data movement, processor architecture, and the implementation used on the target sensor or edge platform. The four learning methods are trained and tested on the same group-aware experimental partitions. Their performance is compared using accuracy, precision, recall, F1-score, ROC-AUC, false-positive rate, confusion matrices, and threshold-related behavior. This enables evaluation of both overall classification performance and the trade-off between detecting clone nodes and incorrectly flagging legitimate sensor activity. Mathematical notation is defined at first use in the equations and accompanying text.

### 3.5. Model Training and Evaluation

The deep-learning models process the prepared behavioral feature representation using one-dimensional convolutional layers. These layers learn patterns across the input characteristics, while nonlinear activation functions allow more complex relationships to be represented. Pooling and fully connected layers are used to combine the learned information for classification, and dropout is applied during training to reduce overfitting. The final output layer uses a sigmoid activation function to estimate the probability that an observation belongs to the clone-node class. The two neural-network models are trained as binary classifiers using Binary Cross-Entropy loss and the Adam optimizer. Their performance is monitored using a separate validation set, and early stopping is applied when the validation loss no longer improves.

Table 1 summarizes the common experimental configuration used to keep the four classifiers comparable, including dataset size, grouping strategy, scaling, optimization, thresholding, and evaluation measures.

Logistic Regression and Random Forest are trained using the same group-aware training data, allowing all four models to be evaluated using a common independent test set.

For classification, each model assigns an observation to either the legitimate or clone-node class based on its learned decision function. For probability-based classification, a default threshold of 0.50 is used. Threshold sensitivity is also examined for the deep-learning models to understand the relationship between clone detection and false-positive behavior. Performance is evaluated using accuracy, precision, recall, F1-score, ROC-AUC, false-positive rate, and confusion-matrix analysis.

The experimental design therefore provides a common behavioral feature representation for comparing conventional machine-learning and deep-learning approaches. Logistic Regression, Random Forest, 1D-CNN, and correlation-enhanced DCNN are evaluated under the same data-partitioning conditions. This helps the study to compare their classification performance, clone-detection sensitivity and false-positive behavior without assuming that any particular learning approach is more effective compared to others.

## 4. Results

**Figure 2:**
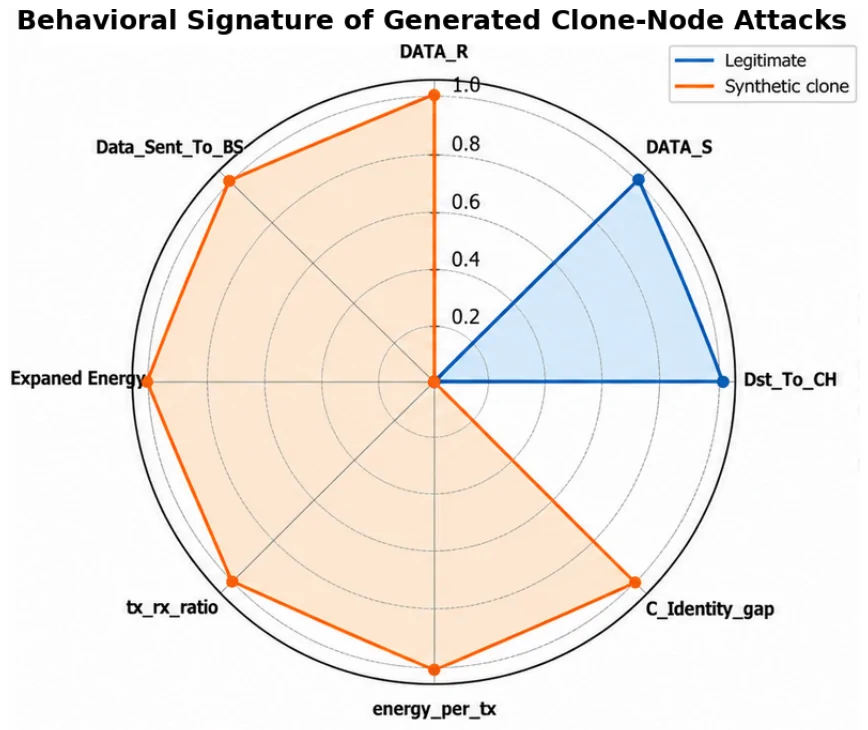
Behavioral differences between legitimate and clone-node observations in the WSN-DS dataset.

Figure 2 presents the behavioral differences between legitimate and synthetic clone-node observations in the WSN-DS dataset. Since WSN-DS does not contain a native clone-node attack class, the clone observations were constructed by preserving the identity of legitimate nodes while introducing controlled changes in communication activity, cluster-head association, traffic behavior, distance, and energy-related characteristics. This represents the basic behavior expected in a node-replication scenario, where the same identity may appear with operational characteristics that differ from those of the original sensor.

Figure 2 shows noticeable differences between legitimate and synthetic clone-node observations across several behavioral characteristics. In particular, differences can be observed in cluster-head identity, transmission activity, traffic-energy behavior, and expended energy. These observations indicate that clone-node activity cannot be represented adequately by a single characteristic. Instead, the combined behavior of several communication, topology, traffic, and energy-related variables provides a more useful representation for intelligent detection. The result therefore supports the use of multi-feature behavioral analysis before applying the different machine-learning and deep-learning classifiers.

The significance of Figure 2 is that it provides experimental evidence for the constructed clone-node scenario before classification is performed. The observed changes demonstrate that identity replication can be represented through measurable variations in sensor behavior, providing a common feature basis for evaluating Logistic Regression, Random Forest, 1D-CNN, and correlation-enhanced DCNN under the same experimental conditions.

**Figure 3:**
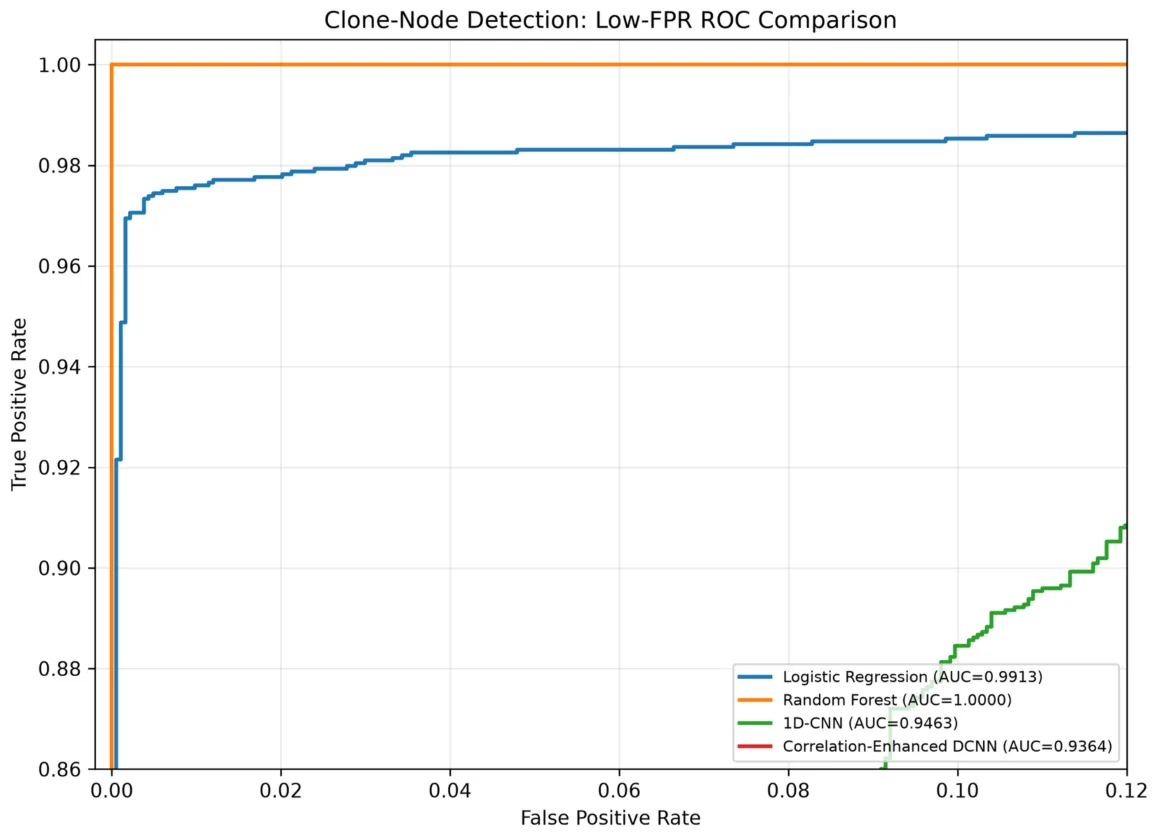
ROC curve comparison of machine-learning and deep-learning models for clone-node detection.

Figure 3 compares the Receiver Operating Characteristic (ROC) curves of the four evaluated models. Random Forest achieved the highest ROC-AUC of 1.0000, followed by Logistic Regression with 0.9913. The 1D-CNN and correlation-enhanced DCNN achieved ROC-AUC values of 0.9463 and 0.9364, respectively. These results show that all four approaches are capable of distinguishing legitimate and synthetic clone-node observations, although their discrimination ability differs considerably.

Random Forest and Logistic Regression show particularly strong separation in the controlled experimental dataset. The result suggests that the behavioral features generated for this scenario contain substantial class-discriminative information that can be effectively captured using conventional machine-learning methods. The deep-learning models also identify clone-related patterns, but the present results show that increased architectural complexity does not automatically lead to better overall discrimination. Similar observations have been reported for structured and tabular classification problems, where conventional learning methods can remain highly competitive when the available features provide clear class separation. Therefore, the ROC analysis supports comparative model evaluation rather than assuming that one class of learning algorithm will always perform best.

**Figure 4:**
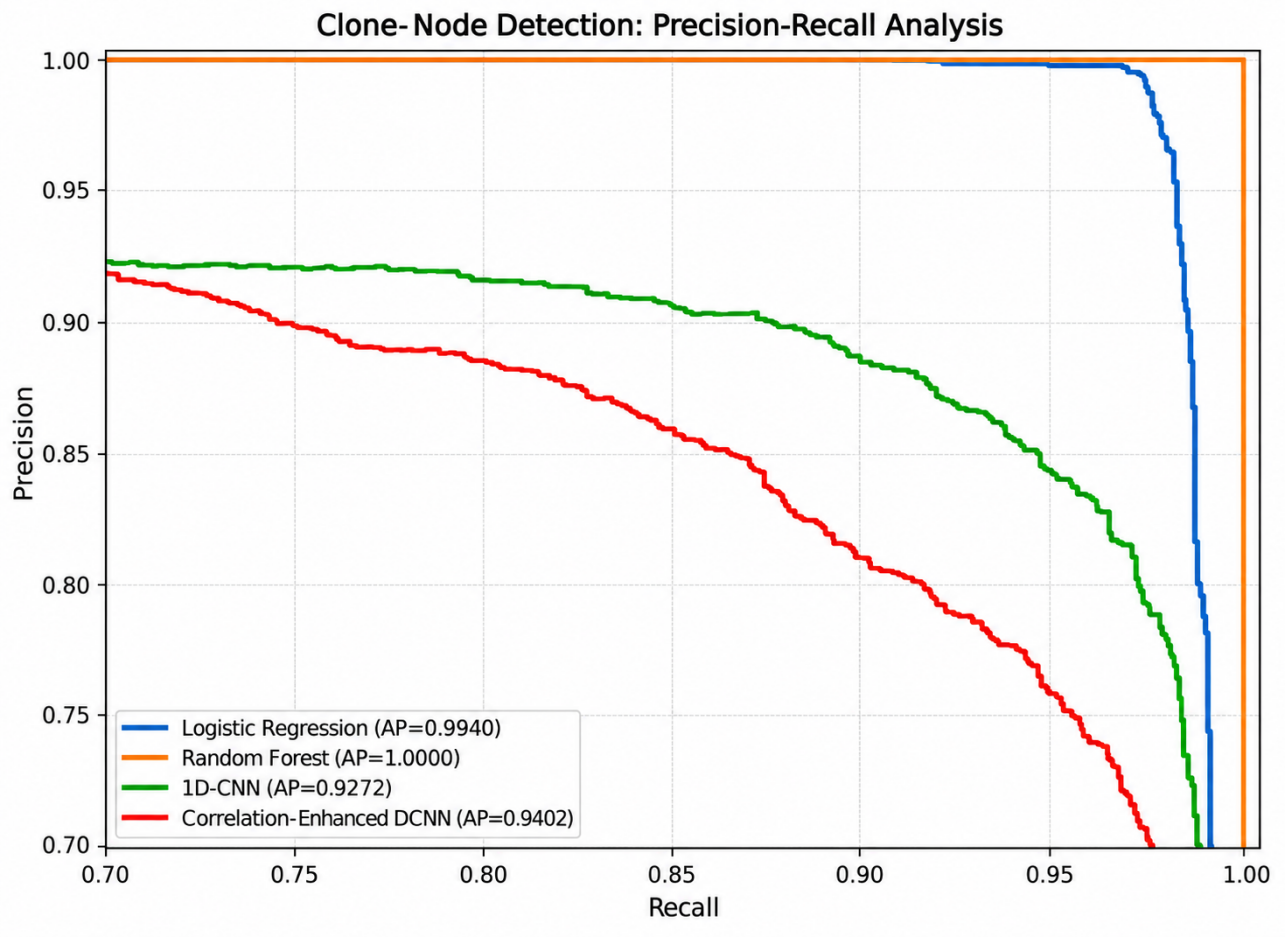
Precision–Recall curve comparison of the evaluated models for clone-node detection.

Figure 4 presents the precision-recall behavior of the evaluated models. Random Forest achieved 100% precision and 100% recall in the controlled test scenario. Logistic Regression also performed strongly, with 99.83% precision and 96.84% recall. The 1D-CNN achieved a recall of 98.91% with a precision of 69.69%, while the correlation-enhanced DCNN reached the highest recall among the deep-learning approaches at 99.46%, although its precision decreased to 60.83%.

These findings illustrate an important difference in the behavior of the evaluated classifiers. Random Forest exhibits a good trade-off between the detection of clone nodes and the correct detection of legitimate observations in the controlled dataset. The two convolutional models are in comparison very sensitive to clone-node activity, but produce a higher number of false alarms. This highlights the need to evaluate the precision and recall together and not with the accuracy. In security applications, high recall is useful as missed attacks may remain active in the network, but too many false positives can increase overhead in verification, communication and node management.

**Figure 5:**
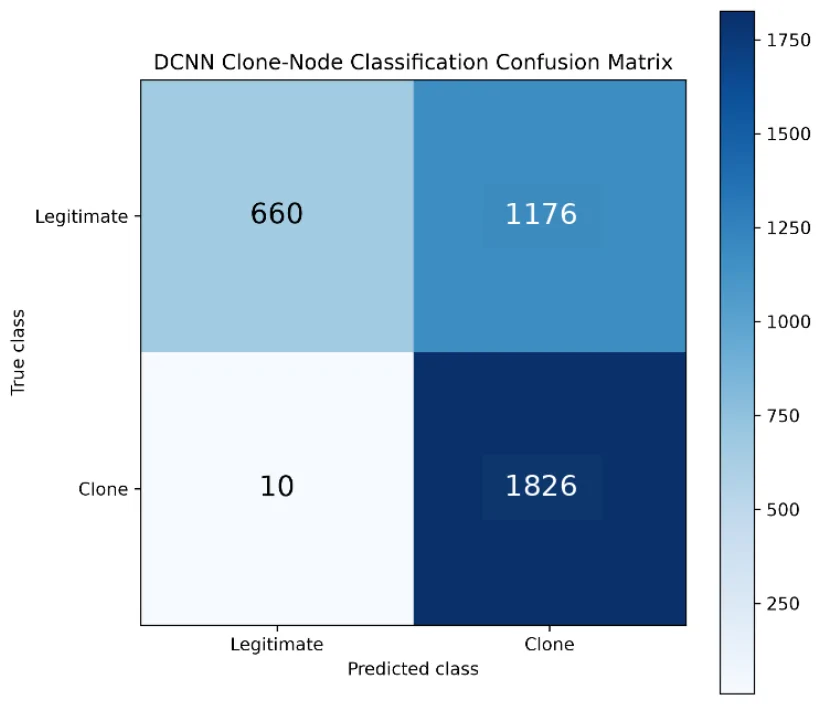
Confusion matrix of the correlation-enhanced DCNN for clone-node detection.

Figure 5 provides a more detailed view of the classification behavior of the correlation-enhanced DCNN. Among the 1836 clone-node observations in the independent test set, the model correctly identified 1826 as clones and incorrectly classified only 10 as legitimate. This corresponds to a recall of 99.46%, demonstrating that the model is highly sensitive to the behavioral changes introduced in the synthetic clone-node scenario. Such high recall is relevant in WSN security because an undetected replica may continue participating in network communication and may potentially affect routing or sensor information.

At the same time, the confusion matrix reveals the main trade-off associated with the DCNN. Of the 1836 legitimate observations, 660 were correctly recognized while 1176 were incorrectly classified as clone nodes. The model therefore favors attack sensitivity over conservative classification. This explains the very high recall of the DCNN, although it has lower accuracy and precision. The result should not be seen as evidence of overall superiority, but as a different detection behavior where the model minimizes missed clone observations at the expense of a relatively high false-positive rate.

This is useful because it highlights a particular area in which convolution-based clone detection could be improved. Better threshold calibration, feature refinement, cost-sensitive learning or alternative network architectures may help to reduce false positives while maintaining the high clone detection sensitivity observed in the current experiment.

**Figure 6:**
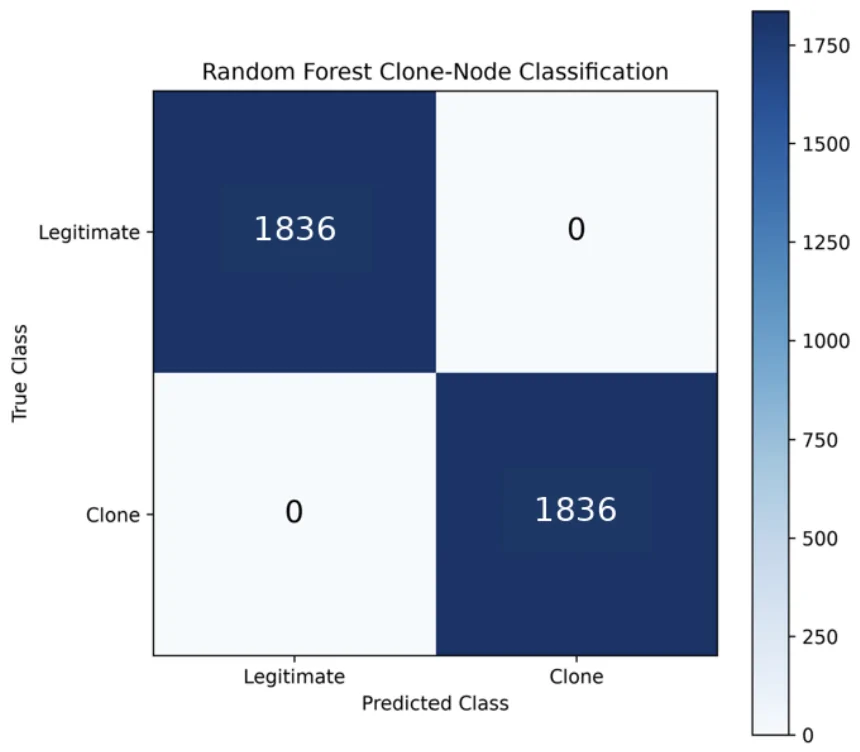
Confusion matrix of the Random Forest model for clone-node detection.

Figure 6 shows the confusion matrix of Random Forest under the same independent test condition. Compared to the DCNN result, Random Forest correctly classified all 1836 legitimate observations and all 1836 clone-node observations with no false positives and false negatives.

Random Forest result is an important comparison with Figure 5. Although the correlation-enhanced DCNN develops a highly sensitive decision boundary which flags many legitimate observations as well, Random Forest provides a complete separation of the two classes in the synthetic clone-node scenario. This implies that the behavioral modifications made during clone creation lead to feature patterns that are suitable for decision tree-based partitioning.

The perfect Random Forest result, however, must be interpreted within the limits of the present experimental design. The same degree of separation cannot be presumed for unknown real-world clone behaviors or independently gathered datasets because the clone observations were produced under controlled conditions from valid WSN-DS records. However, the result indicates that standard ensemble learning can establish a good benchmark for the intelligent detection of clone-nodes, and points out the importance of evaluating the models’ appropriateness concerning the structure of the available behavioral data.

**Figure 7:**
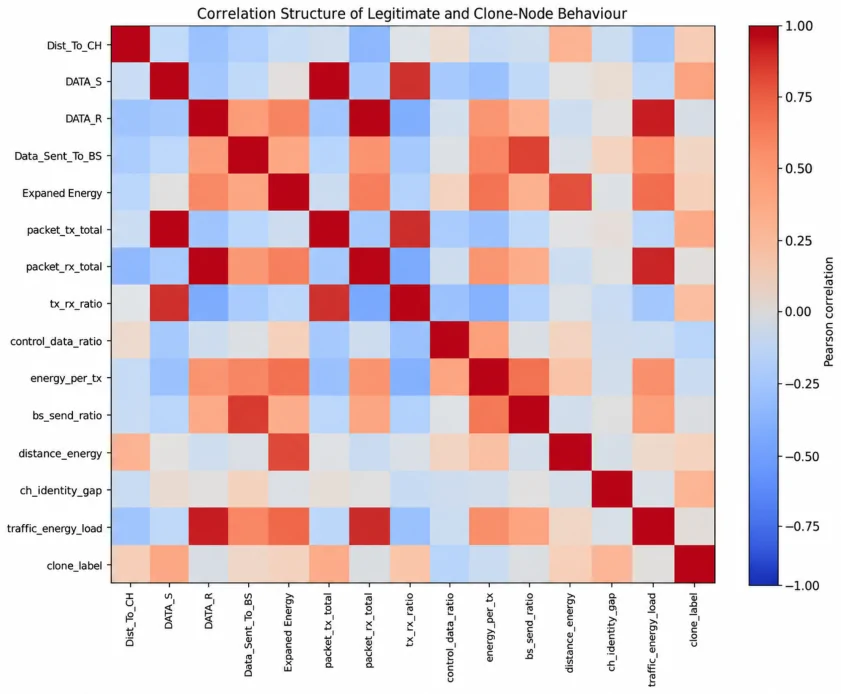
Correlation structure of behavioral features used for clone-node detection.

Figure 7 shows the Pearson correlation structure of the behavioral and derived features used in the clone-node analysis. There are several strong relationships between communication, traffic, distance and energy features. Features of the transmission data are highly correlated with packet transmission behavior. Features of the received data are similarly correlated with packet reception behavior. Traffic and communication features also interact with energy-related features.

The relationship between the clone label and a number of behavioral characteristics is of particular importance. Different degrees of association are found between the clone class and cluster-head identity, transmission activity, traffic-energy behavior and other derived network characteristics. At the same time, no one variable fully describes clone-node behavior. This supports the use of multiple features and their relationships to distinguish replicated nodes from legitimate sensor activity.

Recent sensor-network anomaly research in *Entropy* also shows the value of explicitly modeling relationships among sensor variables and validating the contribution of the relationship-aware component through ablation [25]. The correlation analysis is not only useful for a single classifier, but for the whole intelligent detection framework. The obtained behavioral features can be directly used by conventional models, while the correlation-enhanced DCNN further incorporates feature relationships into its learned representation. Highly correlated variables may contain redundant information, while combinations of moderately related characteristics may uncover behavioral inconsistencies which are not easily apparent when the variables are viewed in isolation. Thus, the observed correlation structure offers a useful foundation for conventional classification and more advanced feature-learning approaches.

**Figure 8:**
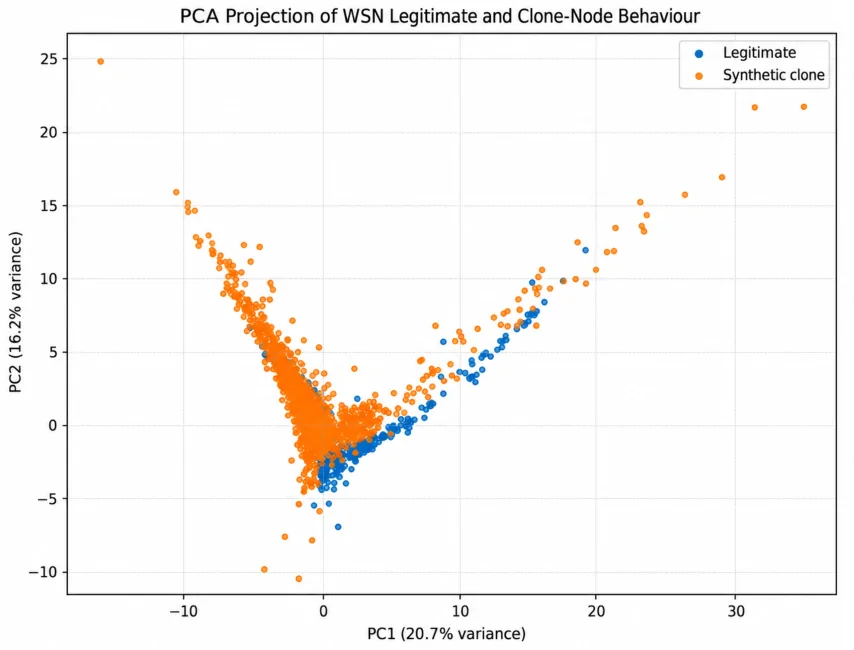
Legitimate and synthetic clone-node behavior in the WSN-DS feature space.

Figure 8 shows legitimate and synthetic clone-node observations in a 2D feature space obtained using Principal Component Analysis (PCA). The first two principal components contribute to 36.8% and 18.9% of the variance, respectively, accounting for about 55.7% of the total variance in the original feature representation.

The PCA visualization reveals that legitimate and synthetic clone-node observations are partially separated in the feature space. The distribution of synthetic clone-node observations is wider, with some samples extending into areas with relatively few legitimate observations. However, there is still a large amount of overlap in the central region. This implies that these two classes are not always separable by a simple linear boundary or a few individual features.

The result of PCA is consistent with the correlation analysis shown in Figure 7. While the correlation matrix describes relationships among individual behavioral characteristics, Figure 8 shows how their combined variation appears in a lower dimensional representation. The partial separation provides additional evidence for the use of multivariate learning methods including both traditional ensemble models and nonlinear neural-network classifiers.

**Figure 9:**
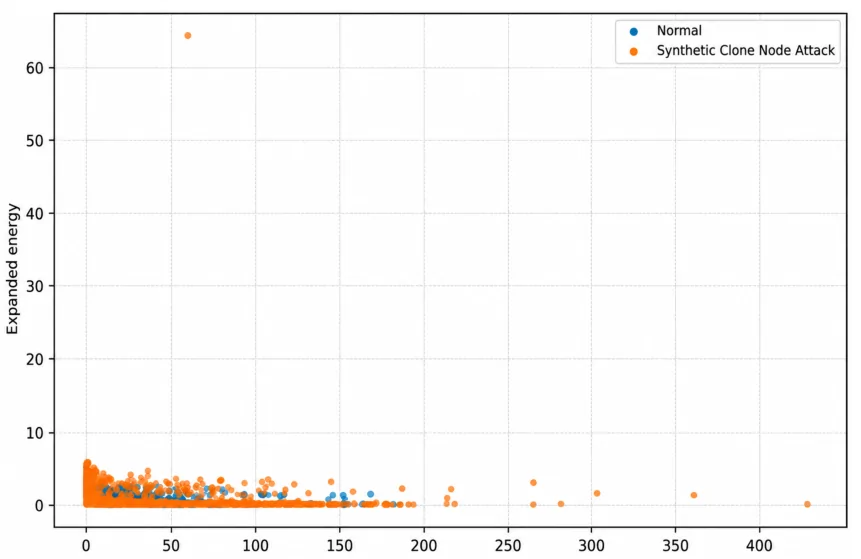
Legitimate and synthetic clone-node behavior—topology–energy behavior.

Figure 9 presents the relationship between cluster-head distance and expended energy for legitimate and synthetic clone-node observations. Legitimate observations are largely concentrated in the lower-distance and lower-energy region, whereas the synthetic clone-node observations show a wider distribution across both characteristics. Several clone observations extend to greater communication distances and higher energy expenditure, indicating behavioral changes introduced by the synthetic clone-node scenario. Although some overlap exists between the two classes, their different distributions support the use of distance and energy characteristics together with other behavioral features for clone-node detection.

The broader distribution shows that node replication can introduce discrepancies between the network position and the energy behavior. However, the overlap between the two classes also shows that neither distance nor energy consumption alone is sufficient for reliable clone-node detection. These characteristics become more informative when they are considered together with communication, traffic, cluster-head, and other behavioral information. Figure 9 therefore reinforces the need for multi-feature intelligent classification rather than a detection rule based on a single sensor property.

**Figure 10:**
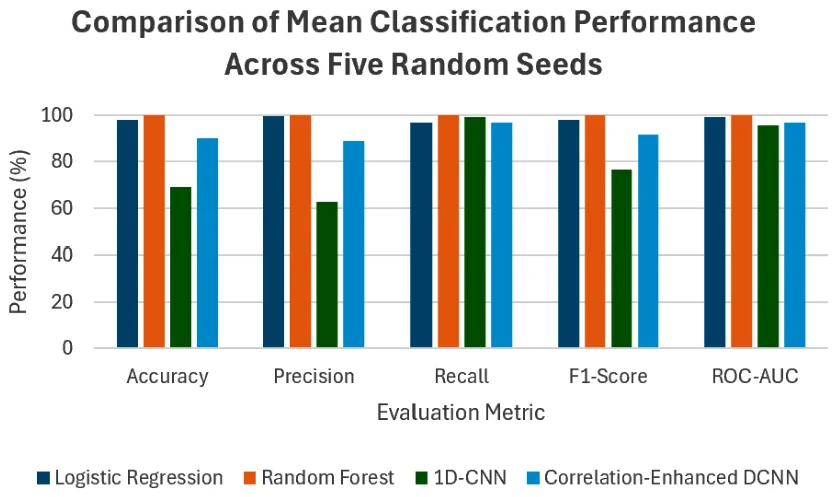
Comparison of mean classification performance across five independent random seeds for Logistic Regression, Random Forest, 1D-CNN, and Correlation-Enhanced DCNN.

Table 2 and Figure 10 summarizes the overall test-set performance. Random Forest achieved the strongest overall classification performance in the controlled experiment, reaching 100% accuracy, precision, recall, and F1-score with an ROC-AUC of 1.0000 and an FPR of 0.00%. Logistic Regression also produced strong performance, achieving 98.34% accuracy, 99.83% precision, 96.84% recall, and 98.31% F1-score, together with a very low FPR of 0.16%.

The two deep-learning approaches showed a different performance profile. The 1D-CNN achieved 98.91% recall but lower accuracy and precision of 77.94% and 69.69%, respectively. The correlation-enhanced DCNN produced the highest deep-learning recall of 99.46%, but its precision of 60.83% and FPR of 64.05% show that this sensitivity is accompanied by a substantial number of legitimate observations being flagged as clones. Random Forest maintains consistently strong performance across the principal evaluation measures, while Logistic Regression also performs well with very low false-positive behavior. The two neural-network approaches remain particularly strong in recall but weaker in precision and false-positive control. These results show that the correlation-enhanced DCNN should not be considered an overall replacement for the conventional classifiers in the present experiment. Its main strength is instead its ability to identify nearly all synthetic clone-node observations.

The plain 1D-CNN versus correlation-enhanced DCNN comparison provides an ablation of correlation-derived input augmentation under the same neural architecture and training configuration. Adding correlation-derived features increased recall from 98.91% to 99.46% and reduced false negatives from 20 to 10. Precision changed from 69.69% to 60.83%, while FPR changed from 43.03% to 64.05%. The ablation therefore indicates that correlation augmentation shifts the operating behavior toward higher clone-node sensitivity, with a corresponding specificity trade-off.

From a research perspective, the comparison demonstrates that greater model complexity does not necessarily produce better overall classification performance. The strong Random Forest result suggests that the behavioral features constructed in the synthetic clone-node scenario contain substantial class-discriminative information that can be captured effectively through ensemble decision trees. Logistic Regression further demonstrates that a comparatively simple model can also perform strongly when the feature representation contains meaningful class separation.

At the same time, the deep-learning results provide a different insight. Both convolutional models show very high clone-node recall, indicating that they are sensitive to behavioral changes associated with replication. The correlation-enhanced DCNN in particular detects 1826 of the 1836 clone observations, but the associated false-positive rate shows that its current decision boundary is overly sensitive. This result provides a useful basis for further work on threshold calibration, feature refinement, cost-sensitive learning and hybrid classification strategies to keep high attack sensitivity but improve false-positive control [14].

Table 2 consolidates the principal test-set metrics. Reading accuracy together with recall and FPR is essential here: the DCNN’s high recall is accompanied by substantially more false alarms, whereas Random Forest and Logistic Regression provide stronger false-positive control in this controlled experiment.

### Repeated-Run and Sensitivity Results

Across five independent seeds (Table 3), Logistic Regression achieved 98.17±0.29% accuracy and 99.23±0.14% ROC-AUC, while Random Forest achieved 99.91±0.07% accuracy and 99.99±0.02% ROC-AUC. The 1D-CNN achieved 68.98±11.81% accuracy, 99.35±0.64% recall, and 95.65±0.99% ROC-AUC. The correlation-enhanced DCNN achieved 90.18±13.44% accuracy, 96.72±3.50% recall, and 96.81±4.11% ROC-AUC. These repeated runs show that the conventional classifiers were highly stable across the tested seeds, whereas the convolutional models, particularly in threshold-dependent accuracy and FPR, were more variable.

The feature-order sensitivity analysis showed that the 1D-CNN is affected by the arbitrary sequence assigned to the engineered tabular features. With the original order, accuracy was 77.94%, recall 98.91%, ROC-AUC 0.9463, and FPR 43.03%. Across five fixed permutations, mean accuracy was 64.51±9.46% (range 55.45–77.83%), mean recall was 99.50±0.66%, mean ROC-AUC was 0.9613±0.0041, and mean FPR was 70.48±19.54%. Thus, ranking performance and clone recall remained comparatively stable, but threshold-dependent accuracy, precision, and false-positive behavior were sensitive to feature ordering. This experiment therefore demonstrates, rather than resolves, the methodological limitation associated with applying local convolution to an unordered tabular feature vector. The 1D-CNN and correlation-enhanced DCNN are consequently interpreted as exploratory comparative architectures in the present setting, and their results are not taken as evidence that one-dimensional convolution is generally appropriate for arbitrarily ordered WSN tabular features.

In the alternate-generator test, the models were trained only on the original generator and evaluated on the separately parameterized held-out clone observations (Table 4). Random Forest retained 96.43% accuracy, 100.00% precision, 92.86% recall, ROC-AUC 0.9907, and 0.00% FPR. Logistic Regression achieved 80.28% accuracy and ROC-AUC 0.8634. The 1D-CNN achieved 71.16% accuracy, 85.35% recall, and ROC-AUC 0.7806, while the correlation-enhanced DCNN achieved 76.01% accuracy, 54.14% recall, and ROC-AUC 0.9007. The retained Random Forest performance under a changed perturbation regime provides evidence that its primary result is not solely a consequence of reproducing the exact original generator parameterization. This conclusion is intentionally restricted to generator-parameter robustness. The alternate generator changes perturbation magnitudes and probabilities but remains within the same general synthetic construction framework; therefore, the experiment cannot establish external validity for independently observed real-world clone attacks.

To quantify uncertainty in the test-set detection proportions, 95% Wilson score confidence intervals were calculated from the reported confusion-matrix counts (1836 legitimate and 1836 synthetic clone-node observations). Logistic Regression detected 1778/1836 clone observations, giving a recall interval of 95.94–97.55%, while its false-positive proportion of 3/1836 corresponds to 0.06–0.48%. Random Forest detected 1836/1836 clones with a 99.79–100.00% recall interval and 0/1836 false positives with an upper 95% bound of approximately 0.21%. The 1D-CNN recall interval is 98.32–99.29% (1816/1836), whereas its FPR interval is 40.78–45.31% (790/1836). For the correlation-enhanced DCNN, the recall interval is 99.00–99.70% (1826/1836) and the FPR interval is 61.83–66.22% (1176/1836). These intervals reinforce the principal interpretation: the deep models are highly sensitive to clone observations, but the DCNN in particular has a statistically large false-positive burden in this synthetic clone-node test set. The intervals complement the point estimates by quantifying uncertainty in the reported test-set proportions.

## 5. Discussion

The results reveal a pronounced trade-off between attack sensitivity and false-alarm control. In particular, the 64.05% FPR of the correlation-enhanced DCNN means that its 99.46% clone recall cannot be considered in isolation. In an operational WSN, such a false-positive burden could trigger unnecessary isolation, re-authentication, routing changes, or operator investigation for legitimate nodes. Threshold calibration, cost-sensitive training, and validation on independently generated or real clone attacks are therefore necessary before deployment claims can be made.

Overall, clone-node detection performance should not be assessed using accuracy alone. The experiments reveal different detection characteristics across the evaluated machine-learning and deep-learning approaches. In the synthetic clone-node scenario, Random Forest provides strong overall classification performance, while Logistic Regression achieves similarly strong discrimination with very low false-positive behavior. In contrast, the convolutional models emphasize clone-detection sensitivity, with both achieving recall above 98%.

The difference between the Random Forest and the correlation-enhanced DCNN is particularly illustrative. Random Forest perfectly separates legitimate and synthetic clone-node observations in the test set, while the DCNN detects almost all clone observations but also falsely flags a significant number of legitimate observations. These different results indicate that the model choice influences not only the overall performance but also the trade-off between missed attacks and false alarms.

Random Forest achieved complete separation on the present synthetic clone-node test partition. This indicates that the multi-feature behavioral representation is highly discriminative for an ensemble tree model under the defined controlled conditions. The alternate-generator analysis provides a narrower robustness result: after training only on the original generator, Random Forest retained 96.43% accuracy, 92.86% recall, and ROC-AUC 0.9907 when perturbation magnitudes and probabilities were changed. This supports robustness to the exact numerical settings of the original generator, but it does not establish external validity. Both the primary and alternate clone observations are generated within the same general synthetic perturbation framework and therefore cannot substitute for independently observed real clone attacks. Accordingly, the manuscript does not infer real-world deployment performance from these results; validation using independently collected clone-attack data or physical WSN testbeds is required before such generalization can be made.

The repeated-seed analysis also distinguishes stable and unstable aspects of the evaluated models. Logistic Regression and Random Forest varied only slightly across the five tested seeds, whereas the neural models showed greater variation in accuracy and false-positive rate. More importantly, the feature-order sensitivity experiment confirms that the 1D-CNN’s threshold-dependent performance changes when identical tabular variables are presented in different arbitrary sequences. Because these engineered features have no natural neighborhood ordering, this dependence is a genuine methodological limitation of local Conv1D processing in the present representation. The permutation experiment is therefore not presented as resolving the issue; it provides empirical evidence of it. Accordingly, the 1D-CNN and correlation-enhanced DCNN should be interpreted as exploratory comparative architectures within this controlled study, not as evidence that convolution is inherently suitable for unordered tabular WSN data. Logistic Regression and Random Forest do not use local convolution across adjacent feature positions and are not subject to this specific feature-adjacency mechanism. Future work should prioritize order-invariant or tabular-specific architectures, or feature groupings whose neighborhood structure is defined independently of arbitrary column order.

Similarly, the lower overall performance of the deep-learning models should not be regarded simply as an unsuccessful result. Their high recall reveals a different security-oriented detection behavior and identifies specific limitations when convolutional architectures are applied to structured WSN behavioral data. In particular, the present results support comparison of model behavior within the controlled synthetic setting, but they do not support a claim that the convolutional architectures are generally suitable for unordered tabular clone-detection data. The findings therefore provide practical guidance for subsequent development, including improved feature selection, probability calibration, cost-sensitive objectives, attention mechanisms, and hybrid approaches that combine the sensitivity of deep models with the strong false-positive control observed in conventional ensemble classifiers.

### Computational Complexity Analysis

The computational requirements of the evaluated approaches differ because conventional machine-learning models and convolution-based models process the behavioral feature space in different ways. Logistic Regression has a relatively simple training and inference structure, while Random Forest requires the construction and evaluation of multiple decision trees. The 1D-CNN and correlation-enhanced DCNN involve additional convolutional, activation, pooling, and dense-layer operations. For this reason, the computational analysis mainly focuses on the neural-network components while recognizing that all four models share the same feature-preparation and data-partitioning stages.

For a one-dimensional convolutional layer, the approximate computational cost depends on the input length, kernel size, number of input channels and number of output filters. The dominant convolutional operation can be expressed approximately as $O(L\times K\times C_{in}\times C_{out})$ where *L* represents the input length, *K* is the convolution kernel size, and $C_{in}$ and $C_{out}$ denote the numbers of input and output channels, respectively [24]. This expression provides an approximate measure of computational growth rather than a direct estimate of execution time on a particular processor.

Training of the neural-network models includes forward propagation, backpropagation, parameter updating, and validation. Early stopping is used to avoid unnecessary epochs when the validation loss no longer improves. Once training is complete, classification requires only a forward pass through the trained model and is therefore computationally less demanding than the training stage [24]. In comparison, Logistic Regression performs a relatively small number of arithmetic operations during inference, while Random Forest evaluates a collection of learned decision paths before combining their outputs.

Behavioral feature construction and correlation analysis add one additional preprocessing stage, which is common for the experimental framework. These operations are carried out on a limited set of sensor characteristics and do not require repeated network-wide optimization during classification. The usefulness of combining learned feature representations with optimization and computational decision methods has also been reported in previous work [10]. However, the main aim of the present study is to perform a comparative and reproducible evaluation of clone-node detection, instead of hardware benchmarking.

Therefore, no specific claims are made regarding inference latency, processor utilization, memory consumption, or real-time edge performance. The implementation details and the sensor/edge hardware used for deployment have a strong impact on how these measures are implemented. In practical WSN environments, computational efficiency is affected not only by arithmetic operations but also by memory access, parameter storage, feature processing, and data movement. These factors are especially important for deep-learning models running on resource-constrained devices.

## 6. Conclusions

This study evaluated behavioral clone-node detection in wireless sensor networks using two conventional machine-learning models and two convolutional neural-network models. The conclusions are restricted to the controlled, behaviorally motivated synthetic clone-node setting used in this study and should not be interpreted as validation on real-world clone attacks. The findings demonstrated that communication, topology, traffic, distance, and energy characteristics can all be applied to distinguish the constructed clone behavior. In the synthetic clone-node scenario, Random Forest performed the best overall, while the correlation-enhanced DCNN detected 1826 out of 1836 clone observations with a high recall of 99.46%. These results highlight the importance of considering both detection sensitivity and false-positive behavior when comparing models for WSN security.

The ablation comparison further clarifies the contribution of correlation-derived information. The correlation-enhanced DCNN increased clone-node recall from 98.91% to 99.46%, demonstrating increased sensitivity to clone behavior, while the plain 1D-CNN maintained a more conservative false-positive profile. Accordingly, correlation augmentation is best interpreted as modifying the sensitivity–specificity balance rather than uniformly improving every metric. Five-seed validation showed particularly stable performance for Logistic Regression and Random Forest, while the alternate-generator evaluation showed that Random Forest retained 96.43% accuracy and 92.86% recall when the numerical perturbation regime was changed. This latter result is interpreted only as robustness to generator parameterization, not as real-world external validity, because both evaluation regimes remain synthetic. The feature-order sensitivity analysis also demonstrated that the 1D-CNN’s threshold-dependent performance depends on the arbitrary ordering of the engineered tabular features. We therefore treat the convolutional models as exploratory comparative architectures rather than as evidence that local one-dimensional convolution is generally appropriate for unordered tabular WSN data. These analyses strengthen the transparency of the evaluation by defining both its robustness and its methodological limits. Future work will prioritize independently collected or testbed-based clone-attack data for external validation and will evaluate order-invariant or tabular-specific architectures, together with threshold calibration, cost-sensitive learning, additional WSN datasets, mobility conditions, and computational measurements on resource-constrained hardware.

## 7. Future Scope

Convolution-based methods require more processing power in return for their capacity to learn more complicated representations, whereas conventional models often offer lower computational complexity during inference. Therefore, lightweight network architectures, smaller feature sets, model compression, and hybrid approaches that combine the high clone-detection sensitivity shown by deep-learning models with the low false-positive behavior seen in conventional models can be investigated in future implementations. Two priorities follow directly from the present limitations. First, external validity should be assessed using independently collected clone-attack observations or controlled physical WSN/testbed experiments rather than additional parameter changes within the same synthetic-generation mechanism. Second, future neural comparisons should emphasize order-invariant or tabular-specific architectures, or explicitly justified feature structures, so that predictions do not depend on arbitrary column adjacency. 

## Figures and Tables

**Table 1:** Experimental and model settings.

Parameter	Setting/Value
Dataset	WSN-DS
Legitimate observations	12,000
Synthetic clone-node observations	12,000
Output classes	Legitimate Node (0), Clone Node (1)
Clone construction	Identity-preserving behavioral modification
Data partitioning	Group-aware train/validation/test split
Feature scaling	Standardization using training-set statistics
Evaluated models	Logistic Regression, Random Forest, 1D-CNN, Correlation-Enhanced DCNN
Convolution type	1D convolution (CNN-based models)
Activation function	ReLU (CNN-based models)
Output activation	Sigmoid (CNN-based models)
Loss function	Binary Cross-Entropy (CNN-based models)
Optimizer	Adam (CNN-based models)
Decision threshold	0.50
Early stopping	Validation-loss based (CNN-based models)
Evaluation measures	Accuracy, Precision, Recall, F1-score, ROC-AUC, FPR

**Table 2:** Model performance comparison.

Model	Accuracy	Precision	Recall	F1	ROC-AUC	FPR
Logistic Regression	98.34%	99.83%	96.84%	98.31%	0.9913	0.16%
Random Forest	100.00%	100.00%	100.00%	100.00%	1.0000	0.00%
1D-CNN	77.94%	69.69%	98.91%	81.76%	0.9463	43.03%
Correlation-Enhanced DCNN	67.70%	60.83%	99.46%	75.49%	0.9364	64.05%

**Table 3:** Five-seed repeated-validation results (mean ± standard deviation).

Model	Accuracy	Precision	Recall	F1	ROC-AUC	FPR
Logistic Regression	98.17±0.29	99.57±0.17	96.75±0.56	98.14±0.30	99.23±0.14	0.41±0.17
Random Forest	99.91±0.07	99.95±0.08	99.88±0.09	99.91±0.07	99.99±0.02	0.05±0.08
1D-CNN	68.98±11.81	62.87±8.71	99.35±0.64	76.70±6.52	95.65±0.99	61.39±24.17
Correlation-Enhanced DCNN	90.18±13.44	88.79±16.76	96.72±3.50	91.80±10.22	96.81±4.11	16.37±27.42

**Table 4:** Performance on the alternate-generator held-out-node test set.

Model	Accuracy	Precision	Recall	F1	ROC-AUC	FPR
Logistic Regression	80.28%	99.73%	60.73%	75.49%	0.8634	0.16%
Random Forest	96.43%	100.00%	92.86%	96.30%	0.9907	0.00%
1D-CNN	71.16%	66.48%	85.35%	74.74%	0.7806	43.03%
Correlation-Enhanced DCNN	76.01%	96.22%	54.14%	69.29%	0.9007	2.12%

## Data Availability

The original contributions presented in this study are included in the article.

## Abbreviations

The following abbreviations are used in this manuscript:

AUC	Area Under the Curve
BCE	Binary Cross-Entropy
CNN	Convolutional Neural Network
DCNN	Deep Convolutional Neural Network
FPR	False Positive Rate
PCA	Principal Component Analysis
ROC	Receiver Operating Characteristic
WSN	Wireless Sensor Network
WSN-DS	Wireless Sensor Network Dataset
